# Hotspot Hunter: a computational system for large-scale screening and selection of candidate immunological hotspots in pathogen proteomes

**DOI:** 10.1186/1471-2105-9-S1-S19

**Published:** 2008-02-13

**Authors:** Guang Lan Zhang, Asif M Khan, Kellathur N Srinivasan, AT Heiny, KX Lee, Chee Keong Kwoh, J Thomas August, Vladimir Brusic

**Affiliations:** 1 Institute for Infocomm Research, 21 Heng Mui Keng Terrace, Singapore 119613; 2 School of Computer Engineering, Nanyang Technological University, Singapore 639798; 3 Department of Biochemistry, Yong Loo Lin School of Medicine, National University of Singapore, Singapore 117597; 4 Department of Microbiology, Yong Loo Lin School of Medicine, National University of Singapore, Singapore 117597; 5 Department of Pharmacology and Molecular Sciences, Johns Hopkins School of Medicine, Baltimore, MD 21205, USA; 6 Product Evaluation and Registration Division, Centre for Drug Administration, Health Sciences Authority, 11 Biopolis Way, #011-03 Helios, Singapore 138667; 7 Cancer Vaccine Center, Dana-Farber Cancer Institute, Boston, MA 02115, USA; 8 School of Land, Crop, and Food Sciences, University of Queensland, Brisbame 4072, Australia

## Abstract

**Background:**

T-cell epitopes that promiscuously bind to multiple alleles of a human leukocyte antigen (HLA) supertype are prime targets for development of vaccines and immunotherapies because they are relevant to a large proportion of the human population. The presence of clusters of promiscuous T-cell epitopes, immunological hotspots, has been observed in several antigens. These clusters may be exploited to facilitate the development of epitope-based vaccines by selecting a small number of hotspots that can elicit all of the required T-cell activation functions. Given the large size of pathogen proteomes, including of variant strains, computational tools are necessary for automated screening and selection of immunological hotspots.

**Results:**

Hotspot Hunter is a web-based computational system for large-scale screening and selection of candidate immunological hotspots in pathogen proteomes through analysis of antigenic diversity. It allows screening and selection of hotspots specific to four common HLA supertypes, namely HLA class I A2, A3, B7 and class II DR. The system uses Artificial Neural Network and Support Vector Machine methods as predictive engines. Soft computing principles were employed to integrate the prediction results produced by both methods for robust prediction performance. Experimental validation of the predictions showed that Hotspot Hunter can successfully identify majority of the real hotspots. Users can predict hotspots from a single protein sequence, or from a set of aligned protein sequences representing pathogen proteome. The latter feature provides a global view of the localizations of the hotspots in the proteome set, enabling analysis of antigenic diversity and shift of hotspots across protein variants. The system also allows the integration of prediction results of the four supertypes for identification of hotspots common across multiple supertypes. The target selection feature of the system shortlists candidate peptide hotspots for the formulation of an epitope-based vaccine that could be effective against multiple variants of the pathogen and applicable to a large proportion of the human population.

**Conclusion:**

Hotspot Hunter is publicly accessible at . It is a new generation computational tool aiding in epitope-based vaccine design.

## Background

The binding of fragments of processed antigens by major histocompatibility complex (MHC) molecules of antigen presenting cells (APCs) and their presentation to T-cells is crucial for immune surveillance and defence against bacteria, parasites, viruses and tumors. Recognition of the MHC-restricted target peptide on the surface of APCs by the surveying T-cells of the immune system is mediated through the T-cell receptors (TCRs) [1,2]. Peptides that are recognized by the TCRs and trigger an immune response are called T-cell epitopes and are essential for initiation and regulation of immune responses. Identification of T-cell epitopes in pathogen proteomes is, therefore, crucial for the design of vaccines and immunotherapies. Mapping of these epitopes experimentally, however, is a challenging task because of the large size of pathogens proteomes [3], great diversity of MHC molecules [4], and the low (~0.1–5%) natural prevalence of T-cell epitopes for a given MHC molecule [5]. The high cost of peptide synthesis, limited access to human peripheral blood samples, and time-consuming experimental assays further add to the challenge. Experimental approaches are therefore combined with a number of prediction tools to screen for candidate MHC binders, putative T-cell epitopes. This combination has dramatically accelerated the process of epitope mapping as the judicious use of the tools enable large number of laboratory experiments to be avoided [6].

It can be postulated that an epitope-based vaccine ideally should contain a minimal number of epitopes that cover a vast majority of the human population [7-10]. T-cell epitopes that promiscuously bind to multiple alleles of a human leukocyte antigen (HLA, human MHC) are prime targets for vaccine and immunotherapy development because they are relevant to larger proportions of the human population. The presence of clusters of promiscuous T-cell epitopes, immunological hotspots, has been observed in several antigens, such as SARS coronavirus nucleocapsid [11], HIV-1 proteins [12-14], and *Chlamydia trachomatis *outer membrane protein [15]. These clusters may be exploited to facilitate the development of epitope-based vaccines by selecting a small number of hotspots that can elicit all of the required T-cell functions [11].

Nearly all existing publicly available promiscuous T-cell epitope prediction servers perform prediction for a single protein sequence per submission, while those that accept multiple sequences are not tailor-made to predict hotspots; they rely on manual visualization techniques. Given the large size of pathogen proteomes, it is painstaking to integrate the individual prediction results, and therefore impractical to use these tools for large-scale systematic study of promiscuous epitopes, which is necessary for a global view of the localizations of the epitopes in the proteome and analysis of their antigenic diversity. Herein, we present Hotspot Hunter, a web-based computational system for large-scale screening and selection of candidate immunological hotspots in pathogen proteomes through analysis of antigenic diversity [9]. It allows screening and selection of hotspots specific to four common HLA supertypes, namely HLA class I A2, A3, B7, and class II DR. The A2, A3 and B7 supertypes together cover approximately 88% of the human population [10,16], irrespective of ethnicity, while class II DR supertype is present in 100% of the population. Hotspot Hunter uses Artificial Neural Network (ANN) and Support Vector Machine (SVM) methods as predictive engines.

It has been reported that combining predictions by several methods results in greater accuracy [17-19]. A soft computing approach was applied to integrate the prediction results produced by both methods used by Hotspot Hunter and the results are presented to users in a succinct and easily understood format. Soft computing is a partnership of several methods, each of them are complementary, not competitive, offering their own advantages to allow solutions to otherwise difficult to solve problems [20]. Importantly, soft computing exploits the tolerance for imprecision, uncertainty and approximation, all characteristics for T-cell epitope data.

## System implementation

The predominant length of peptides that bind HLA class I molecules (HLA-A, -B, and -C) is nine amino acids [21]. HLA class II molecules (HLA-DR) bind longer peptides although through a nine amino acids long binding core [22,23]. The HLA-A2 training dataset had a total of 3050 (675 binders and 2375 non-binders) 9-mer peptides related to 15 variants of the HLA-A2 supertype (0201, 0202, 0203, 0204, 0205, 0206, 0207, 0208, 0209, 0210, 0211, 0214, 0217, 6802 and 6901). The HLA-A3 training dataset had a total of 2216 (680 binders and 1536 non-binders) 9-mer peptides related to eight variants (0301, 0302, 1101, 1102, 3101, 3301, 3303 and 6801). The HLA-B7 training dataset had a total of 4102 (1258 binders and 2844 non-binders) 9-mer peptides related to 13 variants (0702, 1508, 3501, 3502, 3503, 5101, 5102, 5103, 5301, 5401, 5501, 5502 and 5601). These data were mainly from three sources, the MHCPEP database [24], IEDB [25], and published literature, and a set of HLA non-binding peptides (Brusic V., unpublished data). The HLA-A2, -A3 and -B7 datasets are available for download at . The HLA-DR training dataset had 2396 (448 binders and 1948 non-binders) 9-mer peptides related to six HLA-DRB1 variants (0101, 0301, 0701, 0801, 1101 and 1501). The training data was from experiments conducted on 340 15-mers to measure their binding affinity to each of the six HLA-DR alleles. The 15-mers came from three protein sources: MEL40 (aka SSX2, NCBI Accession: AAH16957.1, human melanoma cancer), SSP2 (malaria sporozoite surface protein 2, NCBI Accession: Q01443, *Plasmodium yoelli*), HCV (Hepatitis C virus genome polyprotein, NCBI Accession: P26663). The transformation from 15-mers to 9-mers was performed utilizing SYFPEITHI motifs [26]. Each 15-mer binder was decomposed into overlapping 9-mers which were submitted to SYFPEITHI for HLA-DR binding prediction. The highest scoring 9-mer was considered as a binder and the rest of the 9-mers were not used in the final 9-mer dataset. On the other hand, all overlapping 9-mers decomposed from 15-mer non-binders were considered as non-binders. Since the data originated from a single set of experiments, with relatively consistent experimental conditions, it is reasonable to expect that the DR training dataset is of higher quality relative to datasets aggregated from multiple sources.

The ANN and SVM models employed herein were the same as those used in MULTIPRED1 [27]. Three-layer back propagation networks (267-4-1) with sigmoid activation functions were built for HLA-A2 and -A3 supertype [28]. Training parameters were determined by observing 100 cross-validation runs. The maximum number of the ANN training cycles was set to 300. The values of momentum and learning rate were 0.5 and 0.001, respectively. The training was repeated four times, and four sets of weights were obtained. The final prediction score was the average of the four predictions calculated using the four sets of weights. Three-layer back propagation networks (289-8-1) with sigmoid activation functions were built for HLA-B7 supertype. The maximum number of the ANN training cycles was set to 500. The training was repeated four times, and four sets of weights were obtained. The values of momentum and learning rate were 0.5 and 0.005, respectively. The HLA-DR supertype was built in a similar fashion by a 4-layer back propagation network (268-2-4-1) with a hyperbolic tangent sigmoid activation function between the two hidden layers and a sigmoid activation function between the second hidden layer and the output. The value of momentum was 0.9, whereas the learning rate was not fixed but changed according to the learning process to achieve faster convergence. The initial learning rate was 0.004. In each training epoch, the sum of square errors was compared with that of the previous epoch. If the sum of square errors increased to more than 1.005 times of the previous one, the learning rate was decreased to 0.7 time of the initial learning rate. If the sum of square errors decreased to less than 0.98 times of the previous one, the learning rate was increased to 1.05 time of the initial learning rate. Ten-fold cross-validation results showed that the area under the receiver operating curve (Aroc) for A2, A3, B7 and DR ANN models were 0.83, 0.83, 0.88 and 0.85, respectively.

A SVM with Gaussian kernel (g = 0.1 and c = 0.5) was used for HLA-A2 and Gaussian kernel (g = 0.1 and c = 2) for HLA-A3 supertype [29]. SVMs with two-degree polynomial kernel function were employed for prediction of peptide binding to HLA-B7 and -DR supertype. Ten-fold cross-validation results showed that the Aroc of A2, A3, B7 and DR SVM models are 0.91, 0.95, 0.92, and 0.80 respectively. The selection of architectures, parameter values, activation functions and kernel functions was done through sampling and comparison of performance.

The prediction performances of models for HLA-A2 and -A3 supertypes were validated using experimental results from a systematic study of human papillomavirus type 16 E6 (NCBI Accession: P03126) and E7 (NCBI Accession: P03129) proteins [30]. Two of three HLA-A2 hotspots and all three HLA-A3 hotspots were correctly predicted without false positive [29,31]. Recently, Peters *et al*. [32] conducted a benchmark study to compare the performance of various bioinformatics models in predicting MHC class I binding peptides and reported that our ANN models [33] showed the best performance among external tools in predicting peptides binding to three HLA-A2 supertype alleles (0202, 0203 and 0206) and two HLA-A3 supertype alleles (0301 and 1101). Our SVM models have also been validated using the Peters' datasets. The HLA-A2 SVM model outperformed all the external tools evaluated in [32] and the HLA-A3 SVM model was equal to or better than three of the five HLA-A3 external tools studied [29]. The prediction performances of models for HLA-B7 supertype were validated using experimental results of the tumor-associated antigen survivin (NCBI Accession: NP_001159.2) [34,35]. Hotspot Hunter correctly predicted one hotspot and missed one. The prediction performance for HLA-DR hotspots was validated using experimental results from systematic binding studies of overlapping peptides from myelin oligodendrocyte glycoprotein (MOG) (NCBI Accession: CAA88109) [36] and hepatitis C virus 1B protein (NCBI Accession: AAB00216) [37]. All the predicted hotspots were localized in the experimentally validated hotspot regions. Additional validation study using secretory aspartyl proteinase 2 (Sap2), a major protein which is known to induce immune response during *Candida *infection in human, showed that Hotspot Hunter accurately identified two of the three HLA-DR restricted immunological regions experimentally identified using PBMC proliferation and IL-2 ELISpot assays [38].

Each input protein sequence to Hotspot Hunter is truncated into overlapping 9-mer peptide sequences with an 8 amino acid overlap and analysis is carried out on each individual 9-mer. To predict immunological hotspots (regions of high concentration of 9-mer promiscuous binders), we have developed two scoring schemes for HLA class I and class II supertypes. The scheme for HLA class I supertypes is based on high scoring individual 9-mers within a window of 30 amino acids [31,33], and the scheme for HLA class II supertype is based on average scores of individual 9-mers within a window of 15 amino acids. The selection of window length was based on a trial-and-error based heuristics. Window lengths of 15, 20, 25 and 30 were explored and the results were compared with the representative experimental results [31]. The window length of 30 amino acids was found to be best for class I predictions and the window length of 15 amino acids was best for class II predictions. A region in protein sequence is considered as immunological hotspot specific to a HLA supertype only if its predicted binding strength is above the threshold defined for the supertype. The threshold for each supertype was the sum of the thresholds of corresponding ANN and SVM models, which were selected based on experimental binding data [29,33]. Two flowcharts (Figure S1 and S2) describing the steps involved in identifying hotspots in a single input protein are provided at .

The web interface of Hotspot Hunter is intuitive, making use of a set of simple Graphical User Interface forms. Programs were built using a combination of Perl, CGI and C language. The implementation was carried out in SunOS 5.9 UNIX environment.

## Using the system

**Figure 1:**
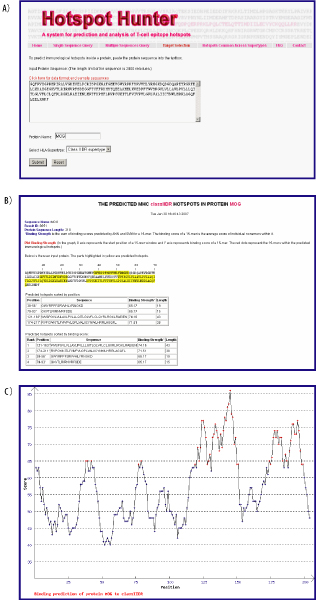
**An example of the input and output pages of Hotspot Hunter when performing "Single Sequence Query"**. The input protein sequence is Myelin Oligodendrocyte glycoprotein (NCBI Accession: CAA88109) and the selected HLA supertype of interest is HLA-DR. A) The input page. B) Prediction result page. C) Plot of predicted scores vs. the amino acid positions in the protein sequence.

Analysis of antigenic regions in a single protein is initiated by opening the "Single Sequence Query" page of Hotspot Hunter, the query protein sequence is pasted into the query box, a name is assigned and the HLA supertype of interest is selected. The input protein sequence should be in FASTA format and at least 15 (for HLA-DR supertype) or 30 (for HLA-A2/A3/B7 supertype) amino acids long and at most 2000 amino acids long. Input sequences that contain symbols other than amino acids (spaces and carriage returns are allowed) or are of lengths outside the acceptable length range, are not be processed – an error message will be displayed. The sequence pasted in the query box is treated as one single protein sequence (carriage returns are ignored). Figure 1 shows the analysis of the human myelin-oligodendrocyte glycoprotein (MOG) for HLA-DR hotspots. The input interface is shown in Fig 1A (numbering is relative to the mature sequence of MOG). Experimental HLA-DR restricted peptides in the mature sequence of MOG (NCBI Accession: CAA88109) formed two hotspots, 73–96 and 121–216 [36]. Hotspot Hunter predicted four hotspots, 38–56, 79–93, 121–163 and 174–211, for the protein (Figure 1B), three of which corresponded to the two experimentally identified regions. The "binding strength" represents the sum of binding scores predicted by ANN and SVM models for each hotspot. The predicted hotspots are displayed in two formats: in ascending order of their positions in the sequence and in descending order of their prediction scores. Users can click on "Plot Binding Strength" on the result page to generate a graph providing a clear overall view of the binding capacity of the input sequence. The X-axis represents the start position of a peptide (window size of 15-amino acids for DR supertype and 30-amino acids for A2, A3 and B7 supertypes) and Y-axis represents the predicted score of the peptide. Figure 1C shows a plot of the binding scores for the MOG protein with each dot (in blue or red color) representing a 15-amino acids peptide. For example, the first 15-amino acid peptide is plotted at position 1 with a score of 63.53. Individual peptides with prediction scores grater than or equal to the threshold 65 for DR supertype are considered as positive binders to multiple DR alleles and are displayed as red dots, while the non-binders below the threshold are displayed as blue dots.

**Figure 2:**
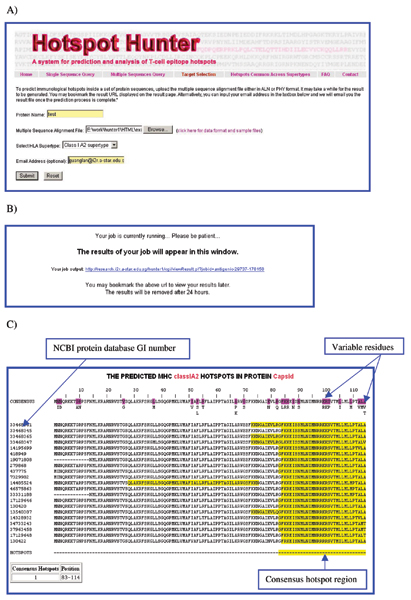
**An example of the input and output pages of Hotspot Hunter when performing "Multiple Sequences Query"**. The input is a set of dengue virus type 1 capsid sequences in ALN (ClustalW) or PHY (PHYLIP) format . A) The input page. B) Waiting message page. C) Prediction results page.

"Multiple Sequences Query" function allows users to predict hotspots in a set of protein sequences by submitting a multiple sequence alignment file either in ALN (ClustalW) or PHY (PHYLIP) format . Predictions are performed on each sequence in the alignment and gaps in the sequences are removed prior to the prediction. However, the gaps are still displayed on the results page and, therefore, hotspots can appear on the results page interspersed by gaps of variable sizes, depending on the alignment. The results page provides a global view of the localizations of the hotspots in the proteome set, enabling analysis of antigenic diversity and shift of hotspots across protein variants. An example of the Hotspot Hunter analysis of a set of dengue virus type 1 capsid sequences is shown in Figure 2. The users may bookmark the URL of the result page (shown in Figure 2B) to access the results which are kept for 24 hours on the server. Alternatively, users can input their email address in the textbox (Figure 2A) and the result file will be emailed to them once the prediction process is complete. The consensus of the input protein sequences is displayed on top of the results page (Figure 2C). The predicted hotspot regions in each protein sequence of the set are highlighted in yellow. Consensus hotspots are defined as hotspots present in at least 50% of the sequences (subject to presence in at least two sequences submitted for prediction) and are displayed at the bottom of the result page. These consensus hotspots are of interest to immunologists as they are shared across multiple protein variant sequences.

**Figure 3:**
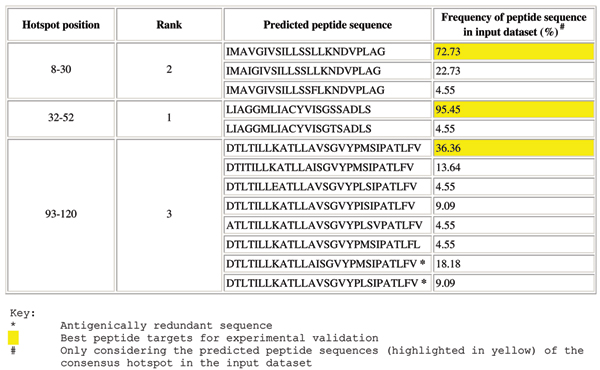
**An example of the output page of Hotspot Hunter when using the "Target Selection" function**. The input is a HLA-DR supertype prediction results page for dengue virus type 1 NS2b sequences in HTML format (output of "Multiple Sequences Query" function). Best peptide targets for experimental validation are highlighted in yellow. Antigenically redundant peptides are peptides whose complete nonamer antigenic diversity is covered by the other peptides at the consensus hotspot position, thus they can be ignored without loss of information on nonamer antigenic diversity among the peptides [9]. Nonamer antigenic diversity was studied because they represent the predominant length of binding cores of T-cell epitopes [21].

"Target Selection" function for multiple sequence queries employs a computational method to identify candidate peptides for the formulation of an epitope-based vaccine that could be effective against multiple variants of the pathogen and is also applicable to a large proportion of the human population. The predicted peptides from consensus hotspots of aligned protein sequences are analyzed for selection of best targets for further experimental validation. All the predicted consensus hotspots are ranked to aid in the decision support, aiming to select the best consensus hotspot. Figure S4 on Hotspot Hunter FAQ page  summarizes the process of decision-making in selecting the best consensus hotspot and best candidate targets. The output of this analysis is a comprehensive report, which facilitates interpretation of results and the selection of validation experiments. A sample of the target selection analysis output for dengue virus type 1 NS2b protein is provided in Figure 3.

Hotspot Hunter performs predictions on four HLA supertypes, HLA-A2, -A3, -B7 and -DR. It allows integration of the prediction results of the four supertypes for identification of hotspots common across multiple supertypes, which is facilitated by the function "Hotspots Common Across Supertypes". The user needs to upload the output files (generated by "Multiple Sequences Query") of the supertypes of interest for the same set of sequences. Hotspots common across any combination of the four supertypes can be analysed.

## Discussion

Experimental approaches for identification of T-cell epitopes are laborious and costly, and thus are not applicable for large-scale screening across multiple HLA alleles and pathogen proteomes. Several publicly available online computational systems have been developed for the prediction of peptides binding to HLA alleles and supertypes, such as SYFPEITHI [26] based on binding motif, BIMAS [39], ProPred1 [40] and PEPVAC [10] based on quantitative matrices, SMM [41] based on stabilized matrix method, MHCPred [42] based on a multivariate statistical method, SVMHC [43,44] based on SVM. Combined computational methods that integrate multiple critical steps of MHC class I antigen processing pathways, such as proteasome cleavage, TAP (transporter associated protein) transport, and MHC class I binding, have been recently proposed as a supporting methodology for prediction of high probability targets for therapeutic peptides and vaccines [45]. Several combined computational models of antigen processing and presentation have been reported, such as NetCTL [46] and MHC-PATHWAY [47]. A major weakness of these methods is that they only represent the major antigen processing pathways; alternative pathways for class I peptide loading exist [48]. For example, proteasomal cleavage is a statistical event with preference for, but not exclusive to, certain cleavage sites. In addition, TAP binding is not the only route for peptide loading into the endoplasmic reticulum (ER); some peptides (signal peptides, membrane peptides, and some viral peptides) are able to access the ER in a TAP-independent manner [49]. Moreover, TAP can transport peptides longer than the optimal HLA-binding length, with endoproteases trimming the peptide to their optimal size in the ER. Taken together, these observations suggest that a simple sequential combination of prediction systems is not adequate. Both relevance and adequacy of such combined systems should be taken with caution. These concerns have been addressed in NetCTL where user can provide the numerical weight for each of the steps in antigen processing and presentation pathway.

Hotspot Hunter is different from other online servers in several aspects. First, the prediction engine combines the strengths of the ANN and SVM methods for better and more robust prediction performance; second, it presents users with predicted immunological hotspots, which are regions of high concentration of predicted promiscuous HLA binding peptides; third, it is suitable for systematic studies of a large set of pathogen proteomes as it can concurrently analyze multiple sequences and present a map providing a global view of their localizations in the proteome, which is the main novelty of the system; and finally, the system provides a utility for selecting candidate experimental targets based on antigenic diversity analysis. Therefore, Hotspot Hunter is a new generation computational tool aiding in epitope-based vaccine design. A customized version of Hotspot Hunter has been integrated into some of our in-house specialized databases, such as the Tumor Antigen Database  and CandiVF – *Candida albicans *Virulence Factor Database [50], for prediction of T-cell epitope hotspots.

## List of abbreviations used

HLA – Human Leukocyte Antigen; MHC – Major Histocompatibility Complex; ANN – Artificial Neural Network; SVM – Support Vector Machine; APC – Antigen Presenting Cell; TCR – T-cell Receptor; NCBI – National Center for Biotechnology Information; MOG – Myelin Oligodendrocyte Glycoprotein; IEDB – Immune Epitope Database and Analysis Resource; TAP – Transporter Associated Protein; ER – endoplasmic reticulum; HCV – Hepatitis C Virus; PBMC – Peripheral Blood Mononuclear Cell.

## Competing interests

The authors declare that they have no competing interests.

## Authors' contributions

GLZ and AMK performed the *in silico *experiments and drafted the manuscript. KNS, ATH, KXL, JTA and VB participated in the design of the study. VB conceived the study, participated in its design and coordination and helped draft the manuscript. JTA, VB and CKK critically reviewed the manuscript. All authors read and approved the final manuscript.
